# Should Physicians Extract Teeth?

**Published:** 1887-06

**Authors:** G. C. Corbin


### Should Physicians Extract Teeth?

BY DR. G. C. CORBIN.

(Read before the Michigan State Dental Society.)

It is a well known fact that grocers often sell sugar for less that its cost at wholesale, and dry goods merchants select some article and do likewise. Such a course may be a cheap mode of advertising. In imitation of this way of doing business a physician in a small town in this State has made it a rule never to charge any of his patients for extracting teeth. On such terms he had a very large business in the line of extracting. His rule has been to extract all teeth that ache. Another physician of the same place for a long term of years carried both turnkeys and forceps for the wholesale extraction of teeth. His price was twenty-five cents, and one could have as many teeth out as he desired. Still a third physician has employed a young man to manufacture cheap rubber plates and has furnished toothless mouths in large numbers. He has advised many to have out all teeth that ache because they ache.

Another physician in this locality habitually carried extracting instruments about the country with him extracting teeth for many reasons. The history of this town in this respect is undoubtedly similar to many towns throughout the country. In consequence of this fact the destruction of teeth has been very great. Judging by the standard and high possibilities of our profession nine mouths out of every ten should possess no plates at all, but should have good natural teeth, yet twenty-five per cent. of the mouths depend upon artificial teeth. For the reasons here stated I ask the question, "Should Physicians Extract Teeth?" It is seldom possible to form a law that will give justice to all parties, and perhaps it would be so still.

It is not to be expected that the general practitioner be a specialist in all points, but because he is not, he should not be at liberty to destroy the eyes, or ears, or teeth. Professional and professing men have been permitted to destroy the eyes, ears, and teeth at liberty, but not the human organization in the aggregate. Had no plate of teeth ever been inserted for less than $100 natural teeth would not have been parted with so recklessly. When all dentists shall become qualified to save teeth, and all patients comprehend the fact, then dentists and teeth shall be estimated by both dentists and patients at their correct value.

DISCUSSION ON DR. CORBIN'S PAPER.

*Dr. Taft.*—If this is the condition of affairs in the enlightened town where the author of this paper has lived, he has hardly done his duty as a missionary. But the fact is that it is too common a thing, and the very practices indicated in this paper, exercised by these persons, is in very many places throughout the country, indulged by members of the dental profession as well. I don't suppose there are any of that sort here. It is sometimes said what in the world are we going to do with so many dentists as are turned out from the colleges in this country. Twenty colleges in the country sending out from five to forty each year, what is to be done with them? I think if we accept the statement of this author there is ample room for all that are likely to be turned out for the next fifty years. If the people of the country are to be redeemed from slaughter there must be a great deal more in the way of correct practice. Now my experience with young men shows too often the first question is, "How can I get ready to make a living?" and this spirit prevails even in some who enter educational institutions. Many think how can I make a living, rather than how can I best prepare myself to serve my fellew men? This should be changed. It is done sometimes, but not always as fully as it ought to be, but for all this I think that those who go out from our institution go with a desire to do good in our profession. It is a lamentable fact that this state of affairs exists with physicians as well as with dentists, and the question occurs to me are the physicians, as a body, any better in this respect than the dentists? My experience in reference to the knowledge of the physicians throughout the country in general is that knowledge in reference to the teeth seems to be very poorly comprehended by most physicians. There are many noble exceptions. A few days ago a case was presented which a physician had been treating and where three leading physicians held a consultation in reference to it. All the teeth in the mouth were out, an abscess opening upon the face opposite the place of the second inferior bicuspid on the left side. That case had been under treatment for three years and was regarded as a malignant tumor, and treated as such. She was informed that it involved a very serious operation and possibly loss of life. The patient presented herself some two or three weeks ago for examination, and the assurance was given her that there was a root of a tooth. This she thought impossible.

The treatment had been external. An appointment was made, and in a few hours an incision was made down through the gum, a little point of the tooth showed itself between the two walls of the process. Clearing it away it was found to be covered with enamel, then it was discovered that it was a supernumerary tooth. It was very firmly fixed. It was a little supernumerary tooth. Within twenty-fonr hours after that the discharge had ceased and there was no pain. The fact is this was a case where the physicians had been wholly mistaken as to the character of the affection. Their statement was that the parts would have to be laid open and a large part of the jaw removed. I give you simply the case and its results. This is only one case of that sort of many. In this case the physicians were not wholly at fault. Three dentists had examined the case and agreed with the physicians. Let us not hastily charge physicians in this matter. They have not made such things a matter of study.

*Dr. Robinson.*--I wish to say a word in favor of the physicians of our town. They are strongly in sympathy with the dentists. There are half a dozen of them. They do not expect to extract teeth and send their work to the dentist. They present their patients to me and we talk the subject over, and decide as to the best way of preserving the teeth. They have confidence in the dentists. This is a sort of a St. Paul business, you must convert them. When they understand that there is something to the dentist beside pulling out the teeth and putting in new sets, they will take you by the hand and say, "How do you do, etc."

I sometimes see a runner of some of the extracting shops and

I ask him "How many teeth have you extracted to-day?" "Were they not good teeth?" "Yes, but the person wished to have a new set and I took them out for two dollars for the first, one dollar for the second, etc." That is another class that is in all the cities of our land. I said that it was impossible to have too many dentists. I have never been afraid of good dentists or physicians. These physicians are not Allopaths or Homeopaths, but friends of the dentists, and I give you my word that if you are men in every sense, you will always find men of this class that are in sympathy with you, and will give you advice on the New Testament plan, without money and without price.

